# José Maria Soares Barata: obituary

**DOI:** 10.1590/S1518-8787.2017051000095

**Published:** 2017-06-13

**Authors:** Eunice Aparecida Bianchi Galati, Delsio Natal, Luiz Roberto Fontes

**Affiliations:** I Departamento de Epidemiologia. Faculdade de Saúde Pública. Universidade de São Paulo. São Paulo, SP, Brasil; II Núcleo de Antropologia Forense. Instituto Médico-Legal. São Paulo, SP, Brasil

On September 5, 2016, the Public Health, and more specifically the Entomology, lost Professor Barata, recognized researcher of Triatominae, subfamily of insects that groups the known “kissing bugs”, among which the vectors of the parasite that causes Chagas disease are present.

Born in Belém, State of Pará, Brazil, he already demonstrated a special affection to insects during his childhood. At the end of his secondary studies, he worked as a technician for the National Department of Rural Endemic Diseases, the institution responsible for combating vectors in Brazil and the precursor of the Superintendence of Public Health Campaigns (SUCAM, a Brazilian public institution responsible for executing public health campaigns), later called the National Health Foundation (FUNASA). With that initial challenge, he learned the basic principles of the anti-vectorial fight, being extremely motivated to embrace this specialty and study the insects involved. He learned the techniques and philosophy of eradication, and he became increasingly interested in the topics of health, enrolling in the Pharmacy and Biochemistry Course of the Universidade Federal do Pará (UFPA). During his undergraduate education, he interned at the Emílio Goeldi Museum, having his first contact with renowned entomologists, who had a decisive contribution in the design of his future. It was in this path that he met Eudina Agar Miranda de Freitas Barata, Professor at UFPA at the time. They married, and this marriage lasted until the end of his days, leaving a wonderful family of four children, eight grandchildren, daughters-in-law, and son-in-law.

**Figure:**
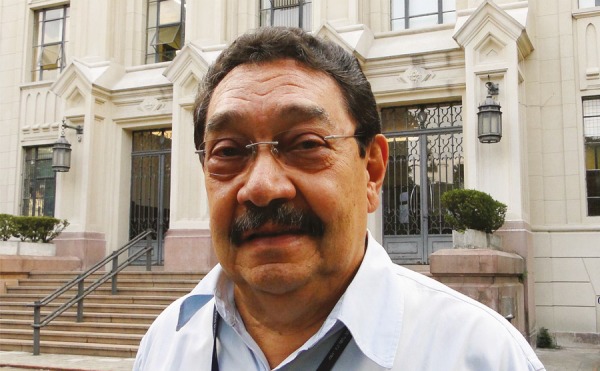
Full Professor José Maria Soares Barata, entomologist and epidemiologist in the Department of Epidemiology of the Faculdade de Saúde Pública of the Universidade de São Paulo, São Paulo, Brazil.

His desire to progress took him to São Paulo, where, in 1970, he attended the Specialization Course in Epidemiological Entomology, of the Faculdade de Saúde Pública of the Universidade de São Paulo, under the coordination of Professor Oswaldo Paulo Forattini. Thus, a strong bond was born with the Institution. Professor Forattini, then head of the Department of Epidemiology, after noting his performance and knowledge on insect vectors, had no doubt and invited him to be part of his team. Then, along with his wife they studied at the Specialization Course in Public Health, which allowed him to expand his knowledge on the complexity of this subject, in the context of interdisciplinarity. Next, he developed his masters and doctorate under the supervision of Professor Forattini and continued in the academic career, reaching the maximum position of Full Professor, in 1999.

Since his coming to the Faculdade de Saúde Pública, he actively participated in the research studies idealized by Professor Forattini, generating numerous studies and publications. In the mid-1970s, the emergence of a new disease was recorded in the Baixada Santista, which spread to the Vale do Ribeira. It was encephalitis, whose agent, a flavivirus, was discovered and described by the team of the Instituto Adolfo Lutz, receiving the name Rocio virus. It was at this time that the works of the Entomology team turned to this region, in order to elucidate the transmission of the disease and contribute to its control or eradication. While Professor Barata performed ecological research studies on Culicidae mosquitoes, for the definition of the vector of that virus, he also participated in studies on Phlebotominae, vectors of *Leishmania* spp., as the region was already characterized by the highest incidence of tegumentary leishmaniasis in the State of São Paulo.

The studies that motivated Professor Barata the most, throughout his career, were on Triatominae, the taxon that groups the kissing bugs, vectors of *Trypanosoma cruzi*, agent of Chagas disease. He always demonstrated his predilection for these insects, among many other entomological challenges. He traveled countless times to Vale do Ribeira, Planalto Paulista, and other destinations in Brazil, which allowed him to gather new information, which he used to produce several publications on the ecology and taxonomy of these hematophagous insects. In this field, he also deepened the study on the external morphology of the eggs of kissing bugs, being one of the pioneers in the use of scanning electron microscopy in Entomology in Brazil, which also allowed him to improve the understanding on the classification and description of kissing bugs. With extensive training in Public Health, it was natural that he was an attentive observer of nature when in the field, always associating the regional landscape and the culture of the population with the epidemiology of the diseases studied.

Another peculiarity was that orchids, especially the native ones – many of them with tiny flowers, which would pass unnoticed by non-sensitive eyes –, fascinated Professor Barata. Part of his leisure time was devoted to the care of these plants, all with identification labels, arranged in a vertical garden in his former residence.

In his academic career, he never abandoned his concern with the formation of persons, having an active part as a professor in the disciplines of Epidemiology offered in the undergraduate, graduate, and specialization courses. In the specialization courses in Medical Entomology, in addition to teaching, he worked extensively as a coordinator. He was zealous in the supervisions and he guided, in all, 18 masters and 13 doctors, several of whom followed the path of the master. He published 80 articles and eight chapters of books that show the richness of the scientific contribution that he gave to the country. His expressive contribution motivated taxonomists to devote two species in his tribute, one triatomine, *Triatoma baratai* Carcavallo & Jurberg, 2000, and one phlebotomine, *Psathyromyia baratai* Sabio, Andrade & Galati, 2015.

Professor Barata was a great enthusiast for the formation of new entomologists. Thanks to his dedication, in the 1990s, the specialization course in Medical Entomology restarted after 10 years. This course was also the basis for the proposal of the Professional Master’s Degree in Entomology in Public Health, started in 2016 and in which, even though he could not be present because of health limitations that plagued him on the threshold of his life, he articulated the substitutes for the disciplines that he would need to teach.

In the individual or team professional production, it is correct to say that he never sought quantity but quality. In the departmental obligations, although averse to management, he did not avoid the teaching issues and he always sought to achieve the goals of the Institution.

His friendly and aggregative nature in human relations was always reflected in his everyday professional work. His daily life was marked by gestures of constant solidarity with colleagues and students, not only from the Faculdade de Saúde Pública, but from several other Institutions. His affable way, still honest if necessary, aroused the admiration of all around him. His strong political views did not lead him away from companionship, being everyone’s friend, even his opponents, from who he never felt hurt and never sought to fight back the professional or personal issues. With humility, simplicity, and affection for all, the doors of the room and the laboratory of Professor Barata were always open to those who were looking for him. If he was busy with any activity, he would stop and answer the visitor, because he was always willing to help, without distinction, those who asked for his help. For him, there was no inappropriate student or visitor, as they were always a fellow professional, even if inexperienced or learning. There were many times that we interrupted him and he, always happily, lengthened conversations and invited us to enjoy a cup of coffee. We are going to miss seeing him in the halls of the School, cafes, Cafeteria, or in any other location that he attended. The festivities in his residence were the coronation, where, surrounded by friends, we tasted delicious dishes from Pará prepared by his entomologist wife. Barata, you left us a legacy, marked on the yearning of the professional competence and true friendship! Thank you for your example and for the opportunity to share your eternal sympathy.

