# Erratum

**DOI:** 10.11606/S1518-8787.2017051000095err

**Published:** 2017-10-05

In the article: “**José Maria Soares Barata: obituário**”, published in *Rev Saude Publica*, in 2017, volume 51, number 53, DOI https://doi.org/10.1590/S1518-8787.2017051000095

**Where you read:**

Letter to the Editor

Rev Saude Publica. 2017;51:53 (top of the page and at the end of the item “how to cite”)

https://doi.org/10.1590/S1518-8787.2017051000095

**You should read:**

Obituary

Rev Saude Publica. 2017;51:72 (top of the page and at the end of the item “how to cite”)

https://doi.org/10.11606/S1518-8787.2017051000095

